# Supplementary material for: Auditory Event-Related Potentials in Two Rat Models of Attention-Deficit Hyperactivity Disorder: Evidence of Automatic Attention Deficits in Spontaneously Hypertensive Rats but Not in Latrophilin-3 Knockout Rats
Source: Genes (Basel). 2025 May 30;16(6):672. doi: 10.3390/genes16060672 (PMC12192062; doi:10.3390/genes16060672)
Supplement: Supplementary file 1 [file genes-16-00672-s001.zip › Lphn3 and SHR Supplemental Materials 5-5-2025.pdf]

Supplementary Table S1

KO/WT P1 Amplitude and Latency ANOVA Findings

	Peak Amplitude				*Peak Latency*			
Source	*df*	*F*	η_p^2	*p*	*df*	*F*	η_p^2	*p*
Between-subject effects								
Genotype	1.00	0.03	.001	.863	1.00	0.56	.016	.460
Sex	1.00	0.13	.004	.722	1.00	**4.37**	**.111**	**.044**
Genotype x Sex	**1.00**	**5.11**	**.127**	**.030**	1.00	2.12	.057	.154
Error (Genotype x Sex)	35.00				35.00			
Within-subject effects								
ITI	1.00	0.35	.010	.561	1.00	**14.96**	**.299**	**<.001**
ITI x Genotype	1.00	2.68	.071	.110	1.00	3.75	.097	.061
ITI x Sex	1.00	0.01	<.001	.929	1.00	0.24	.007	.625
ITI x Genotype x Sex	1.00	1.12	.031	.296	1.00	0.11	.003	.738
Error (ITI)	35.00				35.00			
Tone	**2.64**	**35.48**	**.503**	**<.001**	4.00	**8.63**	**.198**	**<.001**
Tone x Genotype	2.64	2.62	.070	.061	4.00	0.60	.017	.662
Tone x Sex	**2.64**	**3.29**	**.086**	**.029**	4.00	1.70	.046	.153
Tone x Genotype x Sex	2.64	0.80	.022	.481	4.00	1.13	.031	.343
Error (Tone)	92.25				140.00			
ITI x Tone	**3.07**	**18.37**	**.344**	**<.001**	4.00	2.41	.064	.052
ITI x Tone x Genotype	3.07	1.27	.035	.289	4.00	0.73	.020	.557
ITI x Tone x Sex	3.07	0.22	.006	.885	4.00	0.56	.016	.689
ITI x Tone x Genotype x Sex	3.07	1.79	.049	.153	4.00	0.16	.005	.958
Error (ITI x Tone)	107.41				140.00			

Note: P-value considered significant at $p \leq .05$ as indicated by bold font.

Supplementary Table S2

KO/WT N1 Amplitude and Latency ANOVA Findings

	Peak Amplitude				Peak Latency			
Source	*df*	*F*	η_p^2	*p*	*df*	*F*	η_p^2	*p*
Between-subject effects								
Genotype	1.00	0.60	.017	.444	1.00	3.00	.079	.092
Sex	1.00	1.13	.031	.294	1.00	0.38	.011	.540
Genotype x Sex	1.00	2.50	.067	.123	1.00	1.03	0.28	.318
Error (Genotype x Sex)	35.00				35.00			
Within-subject effects								
ITI	**1.00**	**12.26**	**.259**	**.001**	1.00	<0.01	<.001	.978
ITI x Genotype	1.00	0.32	.009	.573	1.00	1.02	.028	.320
ITI x Sex	1.00	0.34	.010	.563	1.00	1.28	.035	.265
ITI x Genotype x Sex	1.00	1.61	.044	.213	1.00	2.99	.079	.093
Error (ITI)	35.00				35.00			
Tone	**1.97**	**45.94**	**.568**	**<.001**	4.00	2.22	.059	.073
Tone x Genotype	1.97	2.02	.055	.141	4.00	0.88	.025	.477
Tone x Sex	1.97	0.61	.017	.544	4.00	**2.77**	**.073**	**.030**
Tone x Genotype x Sex	1.97	1.95	.053	.150	4.00	0.71	.020	.586
Error (Tone)	69.077				140.00			
ITI x Tone	**1.98**	**26.94**	**.435**	**<.001**	4.00	1.01	.028	.405
ITI x Tone x Genotype	1.98	1.45	.040	.241	4.00	1.11	.031	.356
ITI x Tone x Sex	1.98	0.42	.012	.657	4.00	1.37	.038	.246
ITI x Tone x Genotype x Sex	1.98	0.66	.019	.518	4.00	2.05	.055	.090
Error (ITI x Tone)	69.17				140.00			

Note: P-value considered significant at $p \leq .05$ as indicated by bold font.

Supplementary Table S3

KO/WT P2 Amplitude and Latency ANOVA Findings

	Peak Amplitude				*Peak Latency*			
Source	*df*	*F*	η_p^2	*p*	*df*	*F*	η_p^2	*p*
Between-subject effects								
Genotype	1.00	0.01	<.001	.921	1.00	0.57	.016	.455
Sex	1.00	0.11	.003	.743	1.00	**5.61**	**.138**	**.024**
Genotype x Sex	1.00	3.45	.090	.072	1.00	0.22	.006	.646
Error (Genotype x Sex)					35.00			
Within-subject effects								
ITI	1.00	1.70	.046	.201	1.00	**10.96**	**.238**	**.002**
ITI x Genotype	1.00	0.67	.019	.419	1.00	0.05	.001	.828
ITI x Sex	1.00	0.09	.002	.769	1.00	0.37	.010	.548
ITI x Genotype x Sex	1.00	1.20	.033	.281	1.00	0.61	.017	.439
Error (ITI)	35.00				35.00			
Tone	**1.87**	**23.33**	**.400**	**<.001**	4.00	**3.64**	**.094**	**.008**
Tone x Genotype	1.87	2.54	.068	.090	4.00	0.51	.014	.728
Tone x Sex	1.87	1.95	.053	.154	4.00	1.08	.030	.371
Tone x Genotype x Sex	1.87	2.83	.075	.070	4.00	2.40	.064	.053
Error (Tone)	65.41				140.00			
ITI x Tone	**2.49**	**15.48**	**.307**	**<.001**	4.00	1.09	.030	.363
ITI x Tone x Genotype	**2.49**	**3.92**	**.101**	**.016**	4.00	1.02	.028	.397
ITI x Tone x Sex	2.49	0.33	.009	.765	4.00	0.64	.018	.633
ITI x Tone x Genotype x Sex	2.39	0.69	.019	.534	4.00	0.41	.011	.804
Error (ITI x Tone)	140.00				140.00			

Note: P-value considered significant at $p \leq .05$ as indicated by bold font.

Supplementary Table S4

KO/WT N2 Amplitude and Latency ANOVA Findings

	Peak Amplitude				Peak Latency			
Source	*df*	*F*	η_p^2	*p*	*df*	*F*	η_p^2	*p*
Between-subject effects								
Genotype	1.00	1.71	.047	.199	1.00	0.77	.022	.386
Sex	1.00	<0.01	<.001	.987	1.00	1.68	.046	.204
Genotype x Sex	**1.00**	**4.55**	**.115**	**.040**	1.00	3.65	.094	.064
Error (Genotype x Sex)	35.00				35.00			
Within-subject effects								
ITI	1.00	1.04	.029	.316	1.00	0.30	.009	.587
ITI x Genotype	1.00	0.30	.009	.586	1.00	1.16	.032	.288
ITI x Sex	1.00	0.22	.006	.644	1.00	0.04	.001	.853
ITI x Genotype x Sex	1.00	1.98	.054	.168	1.00	1.62	.044	.212
Error (ITI)	35.00				35.00			
Tone	**1.59**	**26.01**	**.426**	**<.001**	**4.00**	**3.41**	**.089**	**.011**
Tone x Genotype	**1.59**	**3.53**	**.092**	**.046**	4.00	0.81	.023	.522
Tone x Sex	1.590	3.12	.082	.063	4.00	1.56	.043	.188
Tone x Genotype x Sex	1.59	1.25	.034	.289	4.00	1.62	.044	.171
Error (Tone)	55.66				140.00			
ITI x Tone	**2.46**	**14.13**	**.288**	**<.001**	4.00	1.13	.031	.345
ITI x Tone x Genotype	2.46	2.22	.060	.104	4.00	0.85	.024	.496
ITI x Tone x Sex	2.46	0.08	.002	.953	4.00	2.13	.057	.080
ITI x Tone x Genotype x Sex	2.46	0.89	.025	.435	4.00	1.47	.040	.216
Error (ITI x Tone)	86.23				140.00			

Note: P-value considered significant at $p \leq .05$ as indicated by bold font.

Supplementary Table S5

KO/WT P1N1 Peak-to-Peak Amplitude ANOVA Findings

Source	*df*	*F*	η_p^2	*p*
Between-subject effects				
Genotype	1.00	0.94	.026	.340
Sex	1.00	0.87	.024	.357
Genotype x Sex	1.00	0.09	.003	.763
Error (Genotype x Sex)	35.00			
Within-subject effects				
ITI	**1.00**	**123.00**	**.778**	**<.001**
ITI x Genotype	**1.00**	**4.41**	**.112**	**.043**
ITI x Sex	1.00	0.51	.014	.478
ITI x Genotype x Sex	1.00	<0.01	<.001	.973
Error (ITI)	35.00			
Tone	**1.39**	**88.08**	**.716**	**<.001**
Tone x Genotype	1.39	0.73	.020	.441
Tone x Sex	1.39	1.23	.034	.289
Tone x Genotype x Sex	1.39	0.76	.021	.430
Error (Tone)	48.64			
ITI x Tone	**2.42**	**64.19**	**.647**	**<.001**
ITI x Tone x Genotype	2.42	1.06	.029	.361
ITI x Tone x Sex	2.42	1.01	.028	.381
ITI x Tone x Genotype x Sex	2.42	1.62	.044	.200
Error (ITI x Tone)	84.65			

Note: P-value considered significant at $p \leq .05$ as indicated by bold font.

Supplementary Table S6

KO/WT N1P2 Peak-to-Peak Amplitude ANOVA Finding

Source	*df*	*F*	η_p^2	*p*
Between-subject effects				
Genotype	1.00	0.58	.016	.453
Sex	1.00	0.87	.024	.357
Genotype x Sex	1.00	0.46	.013	.502
Error (Genotype x Sex)	35.00			
Within-subject effects				
ITI	**1.00**	**17.57**	**.334**	**<.001**
ITI x Genotype	1.00	<0.01	<.001	.950
ITI x Sex	1.00	0.02	<.001	.904
ITI x Genotype x Sex	1.00	0.36	.010	.553
Error (ITI)	35.00			
Tone	**2.66**	**11.92**	**.254**	**<.001**
Tone x Genotype	2.66	0.76	.021	.503
Tone x Sex	2.66	1.45	.040	.236
Tone x Genotype x Sex	2.66	2.65	.070	.060
Error (Tone)	93.04			
ITI x Tone	**64.81**	**4.64**	**.117**	**.005**
ITI x Tone x Genotype	2.85	2.40	.064	.075
ITI x Tone x Sex	2.85	0.20	.006	.884
ITI x Tone x Genotype x Sex	2.85	1.95	.053	.129
Error (ITI x Tone)	99.65			

Note: P-value considered significant at $p \leq .05$ as indicated by bold font.

Supplementary Table S7

KO/WT P2N2 Peak-to-Peak Amplitude ANOVA Findings

Source	*df*	*F*	η_p^2	*p*
Between-subject effects				
Genotype	1.00	2.75	.073	.106
Sex	1.00	0.16	.005	.688
Genotype x Sex	1.00	0.22	.006	.644
Error (Genotype x Sex)	35.00			
Within-subject effects				
ITI	1.00	2.22	.060	.145
ITI x Genotype	1.00	0.10	.003	.753
ITI x Sex	1.00	1.54	.042	.223
ITI x Genotype x Sex	1.00	.05	.001	.830
Error (ITI)	35.00			
Tone	4.00	1.93	.052	.109
Tone x Genotype	4.00	1.11	.031	.353
Tone x Sex	4.00	0.86	.024	.488
Tone x Genotype x Sex	4.00	0.81	.023	.522
Error (Tone)	140.00			
ITI x Tone	4.00	0.44	.012	.779
ITI x Tone x Genotype	4.00	1.18	.033	.322
ITI x Tone x Sex	4.00	2.21	.027	.418
ITI x Tone x Genotype x Sex	4.00	0.63	.018	.646
Error (ITI x Tone)	140.00			

Note: P-value considered significant at $p \leq .05$ as indicated by bold font.

Supplementary Table S8

SHR/WKY P1 Amplitude and Latency ANOVA Findings

	Peak Amplitude				Peak Latency			
Source	*df*	*F*	η_p^2	*p*	*df*	*F*	η_p^2	*p*
Between-subject effects								
Genotype	**1.00**	**10.29**	**.243**	**.003**	1.00	<0.01	<.001	.967
Sex	1.00	0.28	.009	.603	1.00	0.02	<.001	.903
Genotype x Sex	1.00	0.28	.009	.599	1.00	1.35	.041	.253
Error (Genotype x Sex)	32.00				32.00			
Within-subject effects								
ITI	1.00	2.66	.077	.113	1.00	0.27	.008	.606
ITI x Genotype	1.00	0.08	.002	.781	1.00	1.47	.044	.235
ITI x Sex	1.00	1.17	.035	.287	**1.00**	**4.53**	**.124**	**.041**
ITI x Genotype x Sex	1.00	0.22	.007	.642	1.00	3.50	.099	.070
Error (ITI)	32.00				32.00			
Tone	**2.64**	**29.42**	**.479**	**<.001**	**4.00**	**5.37**	**.144**	**<.001**
Tone x Genotype	**2.64**	**6.73**	**.174**	**<.001**	4.00	1.30	.039	.273
Tone x Sex	**2.64**	**5.82**	**.149**	**.002**	4.00	0.57	.018	.684
Tone x Genotype x Sex	2.64	2.00	.059	.127	4.00	0.53	.016	.712
Error (Tone)	84.49				128.00			
ITI x Tone	**2.82**	**5.98**	**.157**	**.001**	4.00	0.44	.014	.774
ITI x Tone x Genotype	**2.82**	**3.00**	**.086**	**.038**	4.00	0.76	.023	.551
ITI x Tone x Sex	**2.82**	**3.15**	**.090**	**.032**	4.00	1.07	.032	.373
ITI x Tone x Genotype x Sex	2.82	0.62	.019	.597	4.00	0.66	.020	.623
Error (ITI x Tone)	90.38				128.00			

Note: P-value considered significant at $p \leq .05$ as indicated by bold font.

Supplementary Table S9

SHR/WKY N1 Amplitude and Latency ANOVA Findings

	Peak Amplitude				*Peak Latency*			
Source	*df*	*F*	η_p^2	*p*	*df*	*F*	η_p^2	*p*
Between-subject effects								
Genotype	1.00	1.71	.051	.201	**1.00**	**5.87**	**.155**	**.021**
Sex	1.00	0.69	.021	.414	1.00	0.03	.001	.876
Genotype x Sex	1.00	<0.01	<.001	.977	1.00	0.13	.004	.723
Error (Genotype x Sex)	32.00				32.00			
Within-subject effects								
ITI	1.00	0.26	.008	.614	1.00	0.37	.011	.547
ITI x Genotype	1.00	0.75	.023	.395	1.00	0.46	.014	.504
ITI x Sex	1.00	2.43	.071	.129	1.00	0.11	.004	.738
ITI x Genotype x Sex	1.00	0.78	.024	.385	1.00	1.95	.058	.172
Error (ITI)	32.00				32.00			
Tone	**3.06**	**13.28**	**.293**	**<.001**	**4.00**	**9.86**	**.235**	**<.001**
Tone x Genotype	3.06	1.68	.050	.174	4.00	1.06	.032	.381
Tone x Sex	3.06	0.59	.018	.629	4.00	0.92	.028	.453
Tone x Genotype x Sex	**3.06**	**3.41**	**.096**	**.020**	4.00	1.71	.051	.153
Error (Tone)	97.86				128.00			
ITI x Tone	**3.05**	**6.23**	**.163**	**<.001**	4.00	0.75	.024	.537
ITI x Tone x Genotype	**3.05**	**3.59**	**.101**	**.016**	4.00	1.91	.056	.113
ITI x Tone x Sex	3.05	0.55	.017	.655	4.00	0.41	.013	.798
ITI x Tone x Genotype x Sex	3.05	0.57	.017	.639	4.00	0.60	.018	.664
Error (ITI x Tone)	97.67				128.00			

Note: P-value considered significant at $p \leq .05$ as indicated by bold font.

Supplementary Table S10

SHR/WKY P2 Amplitude and Latency ANOVA Findings

	Peak Amplitude				*Peak Latency*			
Source	*df*	*F*	η_p^2	*p*	*df*	*F*	η_p^2	*p*
Between-subject effects								
Genotype	1.00	3.63	.102	.066	1.00	0.17	.005	.681
Sex	1.00	1.75	.052	.195	**1.00**	**0.08**	**.776**	**.003**
Genotype x Sex	1.00	0.14	.004	.710	1.00	0.86	.026	.361
Error (Genotype x Sex)	32.00				32.00			
Within-subject effects								
ITI	1.00	0.05	.001	.828	1.00	0.59	.448	.018
ITI x Genotype	1.00	1.47	.044	.234	1.00	0.08	.002	.786
ITI x Sex	1.00	2.10	.062	.157	**1.00**	**0.11**	**.745**	**.003**
ITI x Genotype x Sex	1.00	0.65	.020	.426	1.00	0.02	<.001	.901
Error (ITI)	32.00				32.00			
Tone	**4.00**	**7.29**	**.186**	**<.001**	**3.12**	**7.01**	**.180**	**<.001**
Tone x Genotype	4.00	0.76	.023	.556	3.12	0.87	.027	.461
Tone x Sex	**4.00**	**2.65**	**.077**	**.036**	3.12	0.43	.012	.742
Tone x Genotype x Sex	4.00	2.11	.062	.083	3.12	0.17	.005	.923
Error (Tone)	128.00				99.67			
ITI x Tone	3.00	2.50	.072	.064	3.03	2.21	.065	.091
ITI x Tone x Genotype	3.00	2.21	.065	.092	3.03	0.75	.023	.524
ITI x Tone x Sex	3.00	2.19	.064	.094	3.03	1.08	.033	.360
ITI x Tone x Genotype x Sex	3.0	0.06	.002	.982	3.03	1.45	.043	.233
Error (ITI x Tone)	95.95				96.83			

Note: P-value considered significant at $p \leq .05$ as indicated by bold font.

Supplementary Table S11

SHR/WKY N2 Amplitude and Latency ANOVA Findings

	Peak Amplitude				Peak Latency			
Source	*df*	*F*	η_p^2	*p*	*df*	*F*	η_p^2	*p*
Between-subject effects								
Genotype	**1.00**	**11.54**	**.265**	**.022**	1.00	1.35	.040	.254
Sex	1.00	0.68	.021	.415	1.00	1.87	.055	.181
Genotype x Sex	1.00	<0.01	<.001	.994	1.00	0.17	.001	.899
Error (Genotype x Sex)	32.00				32.00			
Within-subject effects								
ITI	1.00	<0.07	<.001	.932	1.00	1.00	.030	.325
ITI x Genotype	1.00	1.11	.033	.301	1.00	1.40	.042	.245
ITI x Sex	1.00	1.58	.047	.217	1.00	0.03	.001	.857
ITI x Genotype x Sex	1.00	0.60	.018	.445	1.00	0.04	.001	.837
Error (ITI)	32.00				32.0			
Tone	**2.69**	**56.82**	**.640**	**<.001**	4.00	1.33	.040	.262
Tone x Genotype	**2.69**	**4.66**	**.127**	**.006**	4.00	0.91	.028	.459
Tone x Sex	2.69	1.70	.050	.178	4.00	1.11	.033	.355
Tone x Genotype x Sex	2.69	1.53	.046	.216	4.00	0.67	.020	.615
Error (Tone)	86.20				128.00			
ITI x Tone	**2.25**	**7.71**	**.194**	**<.001**	**4.00**	**2.69**	**.078**	**.034**
ITI x Tone x Genotype	2.25	1.46	.044	.239	4.00	0.73	.022	.571
ITI x Tone x Sex	2.25	1.11	.034	.353	4.00	1.75	.052	.144
ITI x Tone x Genotype x Sex	2.25	2.22	.065	.146	4.00	0.21	.007	.930
Error (ITI x Tone)	72.07				128.00			

Note: P-value considered significant at $p \leq .05$ as indicated by bold font.

Supplementary Table S12

SHR/WKY P1N1 Peak-to-Peak Amplitude ANOVA Findings

Source	*df*	*F*	η_p^2	*p*
Between-subject effects				
Genotype	**1.00**	**6.42**	**.167**	**.016**
Sex	1.00	0.54	.017	.468
Genotype x Sex	1.00	0.78	.024	.384
Error (Genotype x Sex)	32.00			
Within-subject effects				
ITI	**1.00**	**39.22**	**.551**	**<.001**
ITI x Genotype	**1.00**	**11.43**	**.263**	**.002**
ITI x Sex	5.52	2.90	.083	.092
ITI x Genotype x Sex	**1.00**	**1.36**	**.252**	**.041**
Error (ITI)	32.00			
Tone	**1.46**	**64.59**	**.669**	**<.001**
Tone x Genotype	**1.46**	**15.68**	**.329**	**<.001**
Tone x Sex	**1.46**	**4.92**	**.133**	**.020**
Tone x Genotype x Sex	1.46	1.80	.053	.183
Error (Tone)	46.59			
ITI x Tone	**2.62**	**33.35**	**.510**	**<.001**
ITI x Tone x Genotype	**2.62**	**13.07**	**.290**	**<.001**
ITI x Tone x Sex	**2.62**	**4.78**	**.130**	**.006**
ITI x Tone x Genotype x Sex	2.62	1.49	.045	.226
Error (ITI x Tone)	83.83			

Note: P-value considered significant at $p \leq .05$ as indicated by bold font.

Supplementary Table S13

SHR/WKY N1P2 Peak-to-Peak Amplitude ANOVA Findings

Source	*df*	*F*	η_p^2	*p*
Between-subject effects				
Genotype	1.00	0.64	.019	.432
Sex	1.00	1.16	.035	.289
Genotype x Sex	1.00	1.12	.034	.298
Error (Genotype x Sex)	32.00			
Within-subject effects				
ITI	1.00	3.19	.091	.083
ITI x Genotype	1.00	0.80	.024	.379
ITI x Sex	1.00	1.01	.031	.322
ITI x Genotype x Sex	1.00	0.65	.020	,427
Error (ITI)	32.00			
Tone	**2.38**	**3.77**	**.105**	**.021**
Tone x Genotype	**2.38**	**5.59**	**.149**	**.003**
Tone x Sex	2.38	2.04	.079	.062
Tone x Genotype x Sex	2.38	2.04	.060	.128
Error (Tone)	75.99			
ITI x Tone	**4.00**	**2.93**	**.084**	**.024**
ITI x Tone x Genotype	4.00	0.88	.027	.479
ITI x Tone x Sex	4.00	1.97	.058	.103
ITI x Tone x Genotype x Sex	4.00	1.61	.048	.177
Error (ITI x Tone)	128.00			

Note: P-value considered significant at $p \leq .05$ as indicated by bold font.

Supplementary Table S14

SHR/WKY P2N2 Peak-to-Peak Amplitude ANOVA Findings

Source	*df*	*F*	η_p^2	*p*
Between-subject effects				
Genotype	**1.00**	**4.47**	**.122**	**.042**
Sex	1.00	0.37	.011	.546
Genotype x Sex	1.00	0.22	.007	.644
Error (Genotype x Sex)	32.00			
Within-subject effects				
ITI	1.00	0.58	.018	,452
ITI x Genotype	1.00	0.94	.029	.339
ITI x Sex	1.00	1.23	.037	.276
ITI x Genotype x Sex	1.00	0.22	.007	.642
Error (ITI)	32.00			
Tone	**4.00**	**38.22**	**.544**	**<.001**
Tone x Genotype	**4.00**	**3.60**	**.101**	**.008**
Tone x Sex	4.00	1.31	.017	.708
Tone x Genotype x Sex	4.00	0.82	.025	.513
Error (Tone)	128.00			
ITI x Tone	**4.00**	**5.99**	**.158**	**<.001**
ITI x Tone x Genotype	4.00	1.06	.032	.377
ITI x Tone x Sex	4.00	1.02	.031	.391
ITI x Tone x Genotype x Sex	4.00	1.65	.049	.167
Error (ITI x Tone)	128.00			

Note: P-value considered significant at $p \leq .05$ as indicated by bold font.
